# Annotations

**Published:** 1901-02-16

**Authors:** 


### Annotations.

### The Report of the Anæsthetics Committee.

When the British Medical Association met in Bournemouth in the year 1891 a committee was appointed to undertake the very important duty of inquiring into the relative safety of the various anæsthetics in common use; and this committee, after presenting several interim reports showing the progress which was being made, has now published its full report, with various very interesting appendices. When we regard the very considerable number of deaths which take place from year to year under anæsthetics, and when we remember the steady extension of the field of surgery, involving as it does the equally steady increase in the number of cases in which the giving of an anæsthetic is required, we see how very important was the work which was thus undertaken. The committee sent out a large number of inquiry forms, collated the answers giving details of nearly 26,000 cases in which anæsthetics were administered during the year 1892, and investigated the circumstances attending a considerable number of deaths which took place under anæsthesia during the same year. The problem set before the committee is one of very great complexity, and perhaps one may fairly say that nothing could better illustrate the extreme folly of many of the dogmatic utterances which we so often hear upon the subject of anæsthetics than the fact that a committee composed of such able men containing within its own circle such an abundance of special knowledge; having such a mass of material before it; and certainly sparing neither time nor trouble in its investigations, should end by finding so many points on which it is unable to speak positively and unanimously. The one point on which the committee seems to speak without hesitation is in regard to the greater danger of chloroform as compared with ether. Another is the conclusion that "by far the most important factor in the safe administration of anæsthetics is the experience which has been acquired by the administrator. In any case the anæsthetisation completely transcends the operation in gravity and importance, and to ensure success—particularly in these cases—it is absolutely essential that an anæsthetist of large experience should conduct the administration." In regard to one matter we must confess that we are disappointed in the report, namely, in the lack of any distinct pronouncement as to the best manner in which chloroform should be administered. It has to be borne in mind that practically the administration of ether means the administration of ether in a particular way. The details may vary, but the principle is the same. When we speak of chloroform, however, the matter is different. This drug is administered in many ways, and in view of the finding of the committee, to the effect that, although ether is "the safest routine agent," circumstances may occur in which it is "both safer and easier" to administer chloroform, the question between the various debated methods of administering chloroform is at least as important, if not more important, than that between chloroform and ether. As to this matter, however, no guide whatever is given except the fairly obvious pronouncement that neither the A.C.E. nor any mixture containing chloroform should be given from a "closed inhaler (*e.g.* Clover's)." We are quite prepared to admit that experiennce goes for much, and that in arranging for the administration of an anæsthetic it is best to call in a specialist. But as the preacher calls not just men but sinners to repentance, so this committee might, we think, have remembered that it was not specialists but outsiders who most required its teaching, and that it would have been well to have spoken somewhat more definitely than it has done in regard to the real questions at issue at the present time.

### The Home Treatment of Lunatics.

One of the most embarrassing problems which our local administrators have to face is that which arises from the yearly increasing number of the insane who have to be provided for in our asylums, and there are some who hold that the tendency, which has of late years manifested itself so strongly, to throw upon the public authorities the whole task of looking after the insane is one which ought to be resisted. In many cases, however, indeed in almost all, in which there is any tendency to violence or excitement, the patient is far better off in a properly managed asylum than among his friends and relatives. It is from the lunatic's point of view that the whole question is regarded by the Lunacy Law, and when people tell us that family ties should not be broken, and family responsibility should not be lightened, we must not forget that ordinary people, even originally kind-hearted people, often make great mistakes, and even perpetrate great cruelties, in the management of their insane relatives. A case which occurred some time ago in the north of London showed that even where the intentions were of the kindest, and where the charge of the lunatic was undertaken at great personal sacrifice, his condition was far less satisfactory than it might have been in an asylum, and a case before the magistrates reported this week, shows how far people will sometimes go in controlling a relative of unsound mind when once they get the idea that physical restraint is necessary. In this case the accused was charged with applying mechanical means of bodily restraint to and ill-treating her lunatic daughter. The alleged ill-treatment of which the prosecution complained, which was testified to by the servants, was that from time to time the patient was confined to bed in a sail-cloth covering of coarse canvas, which was tied down on all sides. There was just room for the head to go through, but underneath the covering she was enveloped in a tight jacket with closed sleeves. There was also webbing tied to the foot of the bed, and it was alleged, moreover, that anklets were fastened on her ankles to prevent her moving her legs. We make no comment on this case as the accused was committed for trial. We merely instance it as an illustration of the sort of "home treatment" of lunacy to which people sometimes resort when they consider that the safety of other members of their family demands it. Whenever restraint is necessary it is best that it should be applied in a properly appointed asylum, unless, indeed, the patient's home can for the time be practically turned into an asylum; which comes to the same thing. It is seldom, however, that people care to face such an expense.

### "Detective" Questions in Life Assurance "Forms."

Many of us when engaged in examining for life assurance, must have wondered by whom the extraordinary "forms" which we are asked to fill up have been contrived, and what can possibly be the meaning of some of the trivial and seemingly absurd questions which abound in them. Dr. T. Glover Lyon,[1] has, however, let the cat out of the bag. He says that, "broadly speaking, the objects of making examination for life assurance may be placed in four divisions:—

1. Detective points regarding the examiner.
2. Detective points regarding the applicant.
3. *Bonâ-fide* information gained from the applicant.
4. *Bonâ-fide* information gained from the examiner."

Here, then, is the explanation of the trivial questions which we find so annoying, and the answering of which is so apt to be slurred over by some examiners. They are "detective" questions—questions put in sometimes to catch a fraudulent applicant, but sometimes to enable the office to judge how the examiner is doing his work. Thus it happens that in American forms "we get repetitions of questions which seem inexplicable, except we remember that they are intended to catch tripping a careless, corrupt, or incapable examiner," which of course is pleasant, and may lead us perhaps to say, "confound their impudence." All the same, it must be remembered that regarding the great mass of the examiners an office can have no knowledge except what comes from the certificates themselves, so that it is well that even the trivial and absurd "detective" questions should be filled up carefully, as only by so doing can proper weight and consideration be secured for the more important statements made in other parts of the report. As for the detective points regarding the applicant, questions relating to the colour of the hair and complexion, and others touching personal peculiarities, they are not, as some might suppose, intended to elucidate abstruse problems as to the life prospects of people possessing such attributes, but are merely put in for purposes of identification. In the same way the questions asked by these offices of the applicant are a sort of cross-examination. A good deal of stress is put upon the answers which the applicant may give: they are even used as a check upon the examiner. "Inconsistent answers lead to a refusal of the policy, and incorrect ones to repudiation of claims." The medical report is but one link in a chain of evidence which has to be adjudicated on by the principal medical officer and the actuary; and any slackness in the answers given to what the examiner thinks unimportant and ridiculous questions is liable to lead to the whole of his evidence being put on one side. This is worth remembering.

[1] Practitioner, Feb. 1901.

### The Nurse Who "Insists."

He would be a bold man who would venture nowadays to call in question the all-importance of the trained nurse in the treatment of disease. She has taken a place from which she will not readily be displaced. Nor would it be well either for the public or the profession that any attempt should be made to disturb the position occupied by the better class of nurse, for nurses of the better class are altogether admirable. Intelligent and well taught, kindly and self-sacrificing, they form the most delightful and trustworthy of helpers. But some of them, and especially some of those who profess to speak in the name of nurses, are not always entirely judicious, and are apt sometimes to forget that after all, notwithstanding their "training," they cannot be more than helpers, strictly limited in regard to their own proper functions, and in all that concerns matters beyond such limits doing only as they are told. That this is so will be admitted at once by all who recognise the superficiality of that glimpse at the great problems of disease which is all that it is possible to give to the average nurse in the course of her training. Nurses are interesting pupils, their curiosity is unbounded, and they always want to know the why and the wherefore. And properly so. The difficulty is to give them some sort of inkling as to this why and wherefore without putting things so plainly and simply that they go away with the idea that they know all about it. Undoubtedly some of them do thoroughly believe in their honest little minds that they do indeed know "all about it;" and then they dogmatise. That is where the trouble comes. Here is a little bit of dogmatism, a laying down of the law such as some nurses love. We take it from an American nursing journal,[1] which says: "There seems but one course for the nurse to pursue in the care of diphtheria cases, viz.: First insist on two negative cultures from the nose and throat of her patient before she believes such a patient free from danger to others, and next insist on the physician in attendance taking cultures from her own nose and throat and giving her the bacteriological report obtained." She is not told to refer the question to the doctor in attendance. She is to make up her own mind on the subject—difficult perhaps, but quite to her taste—and above all things, having obtained from her little books the assurance that "culture examination is always positively correct," she is to "insist" (which she will do *con amore*) on having the culture made, and "insist" on acting upon its indication. A little knowledge is a dangerous thing.

[1] The Trained Nurse.

### The Plague at Hull.

Hull will be very fortunate, and will have much reason to feel satisfied with its sanitary organisation, if in the end it escapes further trouble from its recent smart attack of plague. So far all seems well, the disease not having spread beyond those who were known to have been exposed to infection on the unfortunate ship *Friary*, by which the disease was introduced. But the outbreak has been one of very great virulence. Much will probably now depend upon whether or not the rats in Hull have become infected. Men we can trace, rats we cannot; and as it appears that dead rats were found on board the steamship *Friary* after the vessel left Alexandria, and as a cat which went on board at the latter port showed signs of illness during the voyage, it is much to be feared that the rats on the ship did become infected. If, then, rats left this vessel and fraternised with the shore rats, the trouble may not yet be over. In any case it will be necessary to keep a careful look-out for some time to come before it can be said with certainty that the danger has passed, for the history of epidemics of plague in other places shows that it may continue as a rat disease for a considerable time before it becomes recognised as occurring among the inhabitants of a town

